# Identification of EMT-related alternative splicing event of TMC7 to promote invasion and migration of pancreatic cancer

**DOI:** 10.3389/fimmu.2022.1089008

**Published:** 2023-01-12

**Authors:** Yuanchi Weng, Hao Qian, Liwen Hong, Shulin Zhao, Xiaxing Deng, Baiyong Shen

**Affiliations:** ^1^ Department of General Surgery, Pancreatic Disease Center, Ruijin Hospital, Shanghai Jiao Tong University School of Medicine, Shanghai, China; ^2^ Research Institute of Pancreatic Diseases, Shanghai Jiao Tong University School of Medicine, Shanghai, China; ^3^ Department of Gastroenterology, Ruijin Hospital, Shanghai Jiao Tong University School of Medicine, Shanghai, China

**Keywords:** pancreatic adenocarcinoma, EMT, alternative splicing, TMC7, organoid

## Abstract

**Objective:**

Epithelial-to-mesenchymal transition (EMT) is tightly associated with the invasion and metastasis of pancreatic cancer with rapid progression and poor prognosis. Notably, gene alternative splicing (AS) event plays a critical role in regulating the progression of pancreatic cancer. Therefore, this study aims to identify the EMT-related AS event in pancreatic cancer.

**Methods:**

The EMT-related gene sets, transcriptomes, and matched clinical data were obtained from the MSigDB, The Cancer Genome Atlas (TCGA), International Cancer Genome Consortium (ICGC), and Gene Expression Omnibus (GEO) databases. Key gene AS events associated with liver metastasis were identified by prognostic analysis, gene set variation analysis (GSVA), and correlation analysis in pancreatic cancer. The cell line and organoid model was constructed to evaluate these key gene AS events in regulating pancreatic cancer *in vitro*. Furthermore, we established an EMT-related gene set consisting of 13 genes by prognostic analysis, the role of which was validated in two other databases. Finally, the human pancreatic cancer tissue and organoid model was used to evaluate the correlation between the enrichment of this gene set and liver metastasis.

**Results:**

Prognostic analysis and correlation analysis revealed that eight AS events were closely associated with the prognosis of pancreatic cancer. Furthermore, the expression of TMC7 and CHECK1 AS events was increased in the metastatic lesions of the human tissue and organoid model. Additionally, the knockdown of exon 17 of TMC7 significantly inhibited the proliferation, invasion, and migration of pancreatic cancer cells in 2D and 3D cell experiments. Finally, the expression of exon 17 of TMC17 exhibited a significant correlation with the poor prognosis in pancreatic ductal adenocarcinoma (PDAC).

**Conclusion:**

The AS events of TMC7 and CHECK1 were associated with liver metastasis in pancreatic cancer. Moreover, exon 17 of TMC7 could be a potential therapeutic target in pancreatic cancer.

## 1 Introduction

Pancreatic cancer is a highly malignant gastrointestinal tumor with high recurrence, metastasis, poor prognosis, and a 5-year survival rate lower than 8%. Additionally, most cases of pancreatic cancer are in the progressive stage when diagnosed (1, 2). Although conventional chemotherapy can improve the prognosis of pancreatic cancer patients, chemoresistance is an unavoidable challenge in treating pancreatic cancer (3). Furthermore, it is challenging to identify new therapeutic targets in pancreatic cancer due to the genetic heterogeneity and complicated crosstalk among molecular signaling pathways (4). Therefore, it would be meaningful to identify the critical genes in regulating the occurrence and development of pancreatic cancer while avoiding complex regulatory mechanisms, which would provide a theoretical foundation to develop safer and more effective therapeutic methods for treating pancreatic cancer.

Generally, the human genome contains approximately 20,000 genes. In addition to genetic mutations and post-translational modifications, post-transcriptional modified genes contribute to genetic diversity, such as mRNA alternative splicing (5). Notably, alternative mRNA transcripts can potentially generate protein isoforms with different structures and functionalities (6), such as constitutive splicing, alternative splice site selection (5′ and 3′), intron retention, mutually exclusive splicing, exon skipping, alternative promoter selection, and alternative polyadenylation sites (7). Additionally, alternative splicing also plays an essential role in cancer generation and development, cancer stem cell-like characteristics, angiogenesis, and drug resistance (6–10). Moreover, by analyzing the data from The Cancer Genome Atlas (TCGA) database and setting experiments, Zhang et al. proved that CD44 standard splice isoform can activate the PDGFRβ/Stat3 cascade and induce the cancer stem cell traits, which indicates that CD44 could be a potential target in inhibiting the progress of breast cancer (9). Certainly, there is another gene-spliced variant that could be used as potential therapeutic targets in cancer treatments, such as MDM4, and insulin receptors (11, 12). As the largest cancer gene database, TCGA is characterized by the comprehensiveness in not only the diverse cancer types but also the multi-omics data including gene expression data, miRNA expression data, copy number variation, DNA methylation, SNP, etc. (13). Therefore, TCGA is a suitable candidate in supplying gene alternative splicing involved in carcinogenesis and cancer progression (14). By exploring TCGA and SpliceSeq databases, researchers revealed the aberrant alternative splicing events of DAZAP1, RBM4, ESRP1, and QKI and splicing factors of ESRP1 and RBM5, which contribute to the development of pancreatic ductal adenocarcinoma (PDAC) (15, 16). In addition, Wang et al. also established a six-gene prognostic alternative splicing signature involved in immune cell infiltration in PDAC tissue, which may have far-reaching significance for immunotherapy (17).

The epithelial-to-mesenchymal transition (EMT) of cancer cell indicates that epithelial cancers can transform into various mesenchymal phenotypes, which plays an essential biological role in cancer progression, metastasis, and drug resistance (18). Therefore, the EMT-related gene set is critical when performing the EMT-related analysis. For example, some researchers deduced the pan-cancer EMT signature according to TCGA RNA-seq data and the established EMT markers, such as CDH1 (epithelial marker), CDH2 (mesenchymal marker), VIM, and FN1 (18, 19). Additionally, some researchers even used EMT-related gene sets from the MSigDB database for gene set variation analysis (GSVA) in PDAC (20, 21). However, it is unreliable to use the gene set for other cancers due to the characteristics of PDAC, such as rich mesenchymal components.

In this study, we first performed prognostic analysis to screen EMT-related genes from the MSigDB database and further validated them in another database. Then, we also verify the gene set in paired primary and metastatic PDAC tissues and organoid models. After establishing the EMT-related gene signature, we further screened the important AS events involved in PDAC liver metastasis by analyzing TCGA data and validated using the cell line model and PDAC-derived organoid model.

## 2 Materials and methods

### 2.1 Transcriptomic data and paired clinical data extraction

Transcriptomic data and paired clinical data of PDAC were downloaded from TCGA database (n = 177) and International Cancer Genome Consortium (ICGC) database (Australia cohort, n = 68; Canada cohort, n = 115). Clinical and transcriptomic data of the GSE19280 cohort were obtained from the Gene Expression Omnibus (GEO) database, containing four normal pancreatic tissues, three normal liver tissue, four primary PDAC tissues, and five metastatic PDAC tissues derived from the liver. Finally, log2-transformed gene expression data were used for further analyses.

### 2.2 Tissue collection

We collected fresh PDAC tissues, metastatic PDAC tissue derived from the liver, and adjacent normal tissues from pathologically and clinically diagnosed pancreatic adenocarcinoma patients. All patients signed the informed consent form before participating in the study. The application of these collected tissues was approved by the Institutional Review Board of Ruijin Hospital Affiliated with Shanghai Jiaotong University School of Medicine. The tissue specimens were immediately used for organoid culture or stored in liquid nitrogen until the mRNA extraction. The patients’ information for prognosis analysis is illustrated in **Supplementary Table 1**.

### 2.3 Organoid culture, passage, and transfection

Tumor tissue was minced and digested in Dulbecco’s modified Eagle’s medium (DMEM) containing IV collagenase (Yeasen Biotechnology, Shanghai, China; Cat#40510ES60; 1 mg/ml) and hyaluronidase (Yeasen Biotechnology, Shanghai, China; Cat#20426ES60, 1 mg/ml) at 37°C for 2–4 h. The material was further embedded in growth factor-reduced (GFR) Matrigel (Corning, New York, USA; Cat#356231; working concentration, 1:1) and cultured in human pancreatic cancer complete medium (OuMel, Shanghai, China; Cat#WM-H-05), containing advanced DMEM/F12 medium, WNT pathway agonists, TGF-β pathway inhibitor, BMP pathway inhibitor, and growth factors such as EGF and FGF10. Normal samples were processed with the abovementioned protocol and digested in DMEM containing IV collagenase, hyaluronidase, and soybean trypsin inhibitor (Sigma, Missouri, USA; Cat#T9003; working concentration, 1 mg/ml) for 30 min. For passage, the Matrigel-containing organoid was digested by TrypLE™ Express (Gibco, New York, USA; Cat#; 12604-021; 1×) for 1 h. Then, the sample was centrifuged at 800 rpm for 5 min, and the precipitated cells were embedded in GFR Matrigel and cultured in human pancreatic cancer complete medium or human pancreas organoid complete medium (OuMel, Shanghai, China; Cat#WM-H-04). For transfection, the organoids were processed into single cells. Then, cells were seeded on a 12-well plate covered with GFR Matrigel. On the second day, the cells were transfected by Lipofectamine 2000 (Invitrogen, Carlsbad, CA, USA) for 6 h. Then, the medium was refreshed with a complete medium for 48 h of culture. Finally, cells were collected for subsequent experiments. The siRNA sequences are listed in **Supplementary Table 2**.

### 2.4 RNA-seq analysis and GSVA

TruSeq RNA Sample Preparation kit (Illumina, San Diego, CA, USA) and HiSeq Xten (Illumina, San Diego, CA, USA) were used to construct the RNA-seq library. Gene count was normalized by calculating the transcripts per million (TPM) value. GSVA was performed for pathway enrichment analysis by the “GSVA” package in R 3.5.1.

### 2.5 RNA extraction and qRT-PCR

Total RNA was extracted from the tissues and cells by a TRIzol one-step kit (15596026, Invitrogen, Carlsbad, CA, USA) as previously described (22). The RNA was reversely transcribed into cDNA according to the instructions of PrimeScript RT reagent Kit (RR047A, Takara Bio Inc., Otsu, Shiga, Japan). SYBR Premix EX Taq kit (RR420A, Takara Bio Inc., Otsu, Shiga, Japan) was used for qPCR in 10-μl reaction mixtures in ABI 7500 (ABI, Foster City, CA, USA). The primers were synthesized by Shanghai Sangon Biotech (Shanghai, China), and the sequences are listed in **Supplementary Table 2**. GAPDH was used as an internal reference, and the relative gene expression level was calculated as a 2^−ΔΔCt^ value. Gene relative PSI value level was calculated by dividing 2^−ΔCt^ of the experimental group by 2^−ΔCt^ of the control group.

### 2.6 Cell culture and transfection

Human pancreatic cancer cell lines PATU-8988, BxPC-3, Capan1, MiaCaPa-2, PANC-1, and a normal human pancreatic ductal cell line HPDE were purchased from the cell bank of American Type Culture Collection (ATCC). The cells were cultured in 1640 medium (Gibco, Carlsbad, CA, USA) containing 10% fetal bovine serum in an incubator (Thermo Fisher Scientific Inc., Waltham, MA, USA) under saturated humidity, 5% CO_2_, and 37°C. Before transfection, the cells with 70%–80% confluence were digested with 0.25% trypsin and resuspended in 1640 medium containing 10% fetal bovine serum (FBS). Then, approximately 2 × 10^5^ cells were plated in a 6-well plate. When the cells reached 50%~70% confluence, transfection was performed by Lipofectamine 2000 (Invitrogen, Carlsbad, CA, USA) and incubated for 6 h before the culture medium replacement with 1640 medium containing 10% FBS. After 48 h, cells were collected for subsequent experiments. The siRNA sequences are listed in **Supplementary Table 2**.

### 2.7 Cell migration and invasion assay

Migration and invasion assays were performed according to the previous study (22). Briefly, cells were resuspended in a serum-free medium (10^6^ cells/ml) and seeded on the Transwell chamber (Corning Glass Works, Corning, NY, USA) at a volume of 300 μ/well. Then, 600 μl of medium containing 10% FBS was added to the basolateral chamber. For the invasion assay, the Transwell chamber was covered with 50% Matrigel (BD Biosciences, Franklin Lakes, NJ, USA) mixed with a serum-free medium at a 1:1 volume ratio and then incubated at 37°C for 2 h. After 24 or 48 h of incubation, the Transwell chamber was fixed with 4% paraformaldehyde and stained with crystal violet. Under a high-magnification microscope (×400), five fields were randomly selected to evaluate the average number of invasive and migratory cells. The experiment was repeated in triplicates.

### 2.8 3D organoid invasion and growth assay

The cells separated from the organoid were resuspended in a complete medium to prepare the cell suspension at 2 × 10^4^/ml and mixed with Spheroid Invasion Matrigel at 1:1 (R&D, California, USA). Then, 20-μl mixtures were seeded at a 48-well plate for each well. After 5 days of incubation, the organoids were imaged by a high-magnification microscope (×400).

### 2.9 Statistical analysis

All data were processed by GraphPad Prism 6.0 (GraphPad Prism, RRID : SCR_002798). Student’s t-test was carried out to compare the difference between groups. As for survival analysis, a log-rank test was performed, and the Kaplan–Meier curve was plotted. Heatmap and forest graph were plotted by using the “GSVA” and “forestmodel” packages in R 3.5.1, respectively. Cox univariate analysis was performed by using the “survival” package in R. All results are presented as mean ± 95% CI from three repeats. A p-value of less than 0.05 was considered statistically significant.

## 3 Results

### 3.1 Redefining of EMT signature associated with inferior prognosis in pancreatic cancer

**Figure 1:**
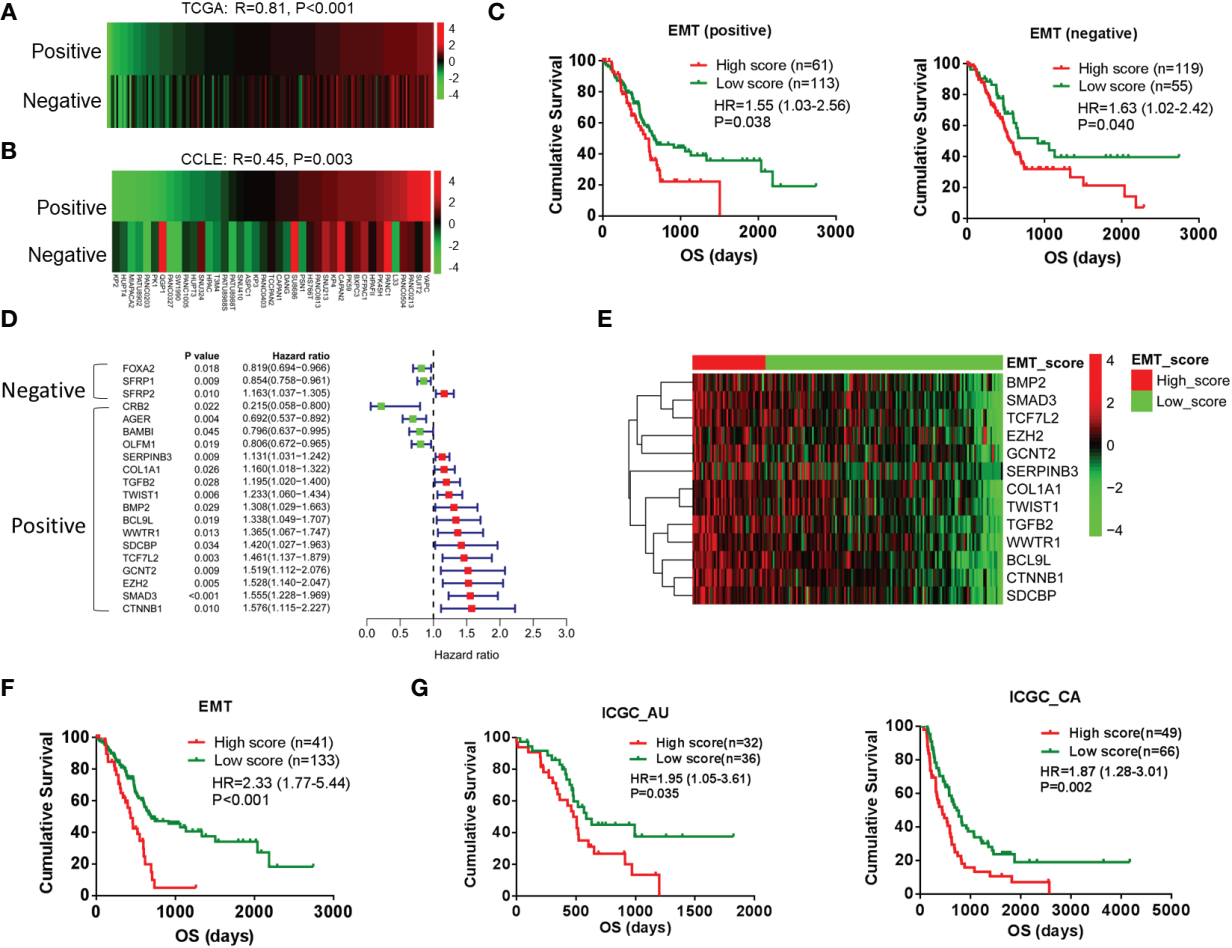
Redefining of EMT signature by prognosis analysis in pancreatic cancer. **(A, B)** Pearson correlation analysis between EMT-related gene sets positively (n = 34) regulating EMT and EMT-related gene sets negatively (n = 23) regulating EMT by using GSVA scores in TCGA (PAAD patients) and CCLE databases (PAAD cell lines). **(C)** Kaplan–Meier analysis of overall survival using GSVA scores of the above two gene sets. **(D)** Forest plot shows the results of univariate Cox analysis of genes in the above two gene sets. **(E)** Heatmap shows the gene expression of 13 EMT-related genes in low-score and high-score groups. **(F)** Kaplan–Meier analysis of overall survival of low-score and high-score groups. **(G)** Kaplan–Meier analysis of overall survival based on the GSVA scores of gene set was performed in other two cohorts in ICGC database. EMT, epithelial-to-mesenchymal transition; GSVA, gene set variation analysis; TCGA, The Cancer Genome Atlas; CCLE, Cancer Cell Line Encyclopedia; ICGC, International Cancer Genome Consortium.

To establish the gene set involved in the EMT of pancreatic cancer, we downloaded the gene set that was involved in positively or negatively regulating the transition from epithelial to mesenchymal from the MSigDB database. First, GSVA was performed by EMT-related gene set positively (n = 34) or negatively (n = 23) regulating EMT in TCGA database. There was a positive correlation between the two gene sets (**Figure 1A**), and the Cancer Cell Line Encyclopedia (CCLE) database (**Figure 1B**). As shown in the prognostic analysis, the two gene sets were positively correlated to poor prognosis (**Figure 1C**). According to the univariate Cox analysis, only 2 of 23 genes were associated with favorable prognosis in the gene set of negatively regulated transition from epithelial to mesenchymal. In contrast, 13 of 23 genes were associated with poor prognosis in the gene set of positively regulated transition from epithelial to mesenchymal (**Figure 1D**). The results indicated that not all EMT-related genes act as we understand in pancreatic cancer. Therefore, these 13 genes with poor prognoses are used to redefine positive EMT-associated genes in pancreatic cancer. As shown in **Figures 1E, F**, the PDAC cases with high EMT scores exhibited poor prognosis by the GSVA and prognosis analysis. Additionally, this gene set exhibited a significant influence on prognosis, according to the other two cohorts in the ICGC database (**Figure 1G**).

### 3.2 EMT signature is significantly associated with liver metastasis

**Figure 2:**
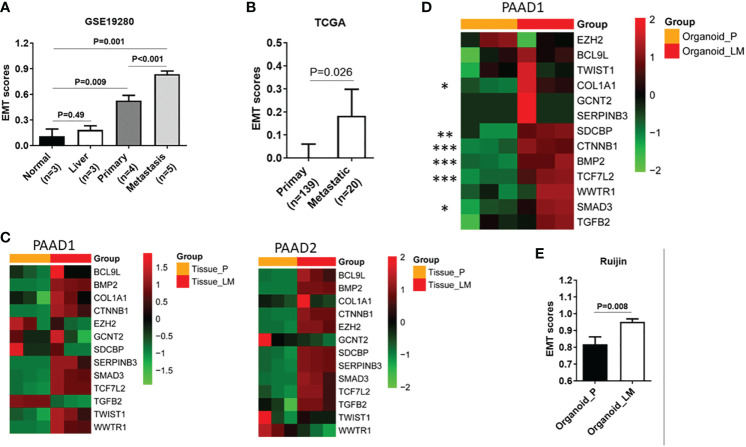
EMT signature is significantly associated with liver metastasis. **(A)** Expression of GSVA scores in normal tissue, liver tissue, primary lesion, and metastatic lesion of PDAC. **(B)** Expression of GSVA scores in primary lesions with distant metastases and primary foci without distant metastases in the TCGA database. **(C)** In our center, heatmap shows expression of 13 genes of EMT-related gene set in primary lesions and metastatic lesions. **(D)** Heatmap shows the expression of 13 genes in the EMT gene set in organoids derived from metastatic lesions and organoids derived from primary lesions. **(E)** Expression of GSVA between organoids derived from metastatic tumor and organoid-derived from primary tumor. EMT, epithelial-to-mesenchymal transition; GSVA, gene set variation analysis; PDAC, pancreatic ductal adenocarcinoma; TCGA, The Cancer Genome Atlas. P value, "*" 0.01-0.05; "**" 0.001-0.01; "***" 0_0.001.

It is well known that EMT is closely related to the invasion and metastasis of tumors. Therefore, we further utilize the dataset GSE19280 for external verification. As shown in the GSVA, the EMT exhibited the most significant characteristics in metastatic lesions, followed by the primary lesion and normal (**Figure 2A**). These results verify the reliability of the EMT-related gene set in predicting PDAC metastasis. According to TCGA database, primary lesions with distant metastases exhibited enhanced EMT signatures compared to those without distant metastases (**Figure 2B**). In our center, metastatic lesions showed almost all evaluated expression of genes in the EMT-related gene set compared to primary lesions (**Figure 2C**). We have successfully constructed relevant primary lesion-derived organoids and metastatic lesion-derived organoids (PAAD 1-6). According to the RNA-seq results, in the EMT gene set, almost all genes showed an increased expression in metastatic lesion-derived organoids (**Figure 2D**). Furthermore, as shown in GSVA, metastatic lesion-derived organoids exhibited enhanced EMT signatures as compared to the primary lesion-derived organoids (**Figure 2E**).

### 3.3 Discovery of gene alternative splicing event associated with EMT in PDAC

**Figure 3:**
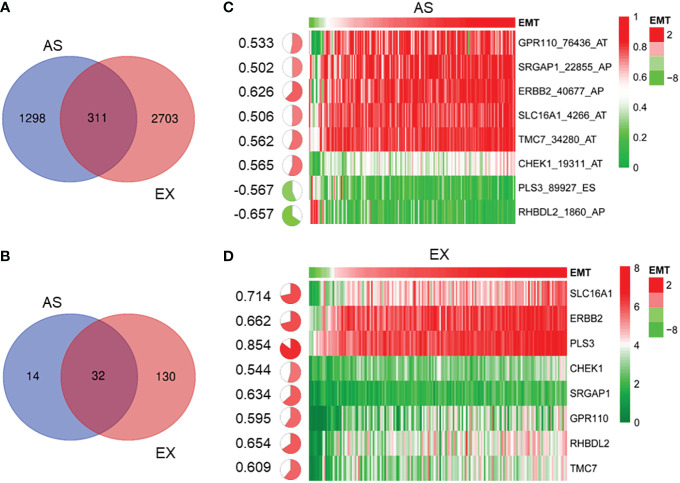
Identification of gene alternative splicing event associated with EMT in PDAC. **(A)** Venn diagram shows 311 genes whose mRNA levels and AS events are associated with prognosis (p < 0.01). **(B)** Venn diagram shows 32 genes whose mRNA levels and AS events were closely related to the EMT signature. **(C)** According to average AS value of between 0.2 and 0.8, heatmap shows that eight gene AS events were significantly related to the GSVA score of EMT **(C)** and eight genes whose mRNA levels were significantly related to the GSVA score of EMT **(D)**. EMT, epithelial-to-mesenchymal transition; PDAC, pancreatic ductal adenocarcinoma; AS, alternative splicing; GSVA, gene set variation analysis.

The functionality of the gene depends on not only the expression level of genes but also the mRNA alternative splicing. Therefore, we performed Cox univariate analysis to explore the genes involved in the interaction among prognosis, mRNA levels, and AS events. As shown in **Figure 3A**, the mRNA levels and AS events of 311 genes were involved in regulating the prognosis by analyzing the intersection (p < 0.01). Furthermore, the correlation between the EMT signature, the mRNA expression of 311 genes, and AS events was analyzed. In 311 genes, the mRNA expression of 32 genes was associated with the AS events and EMT signature, as shown in **Figure 3B**. Generally, the AS values distribute between 0 and 1. To filter genes, the genes with AS values between 0.2 and 0.8 were selected for the following analysis. Finally, 8 of 311 genes were confirmed, as shown in **Figure 3C** (AS event was significantly related to the GSVA score of EMT) and **Figure 3D** (gene expression was significantly related to the GSVA score of EMT).

### 3.4 Screening and identification of gene AS events associated with liver metastases of pancreatic cancer

**Figure 4:**
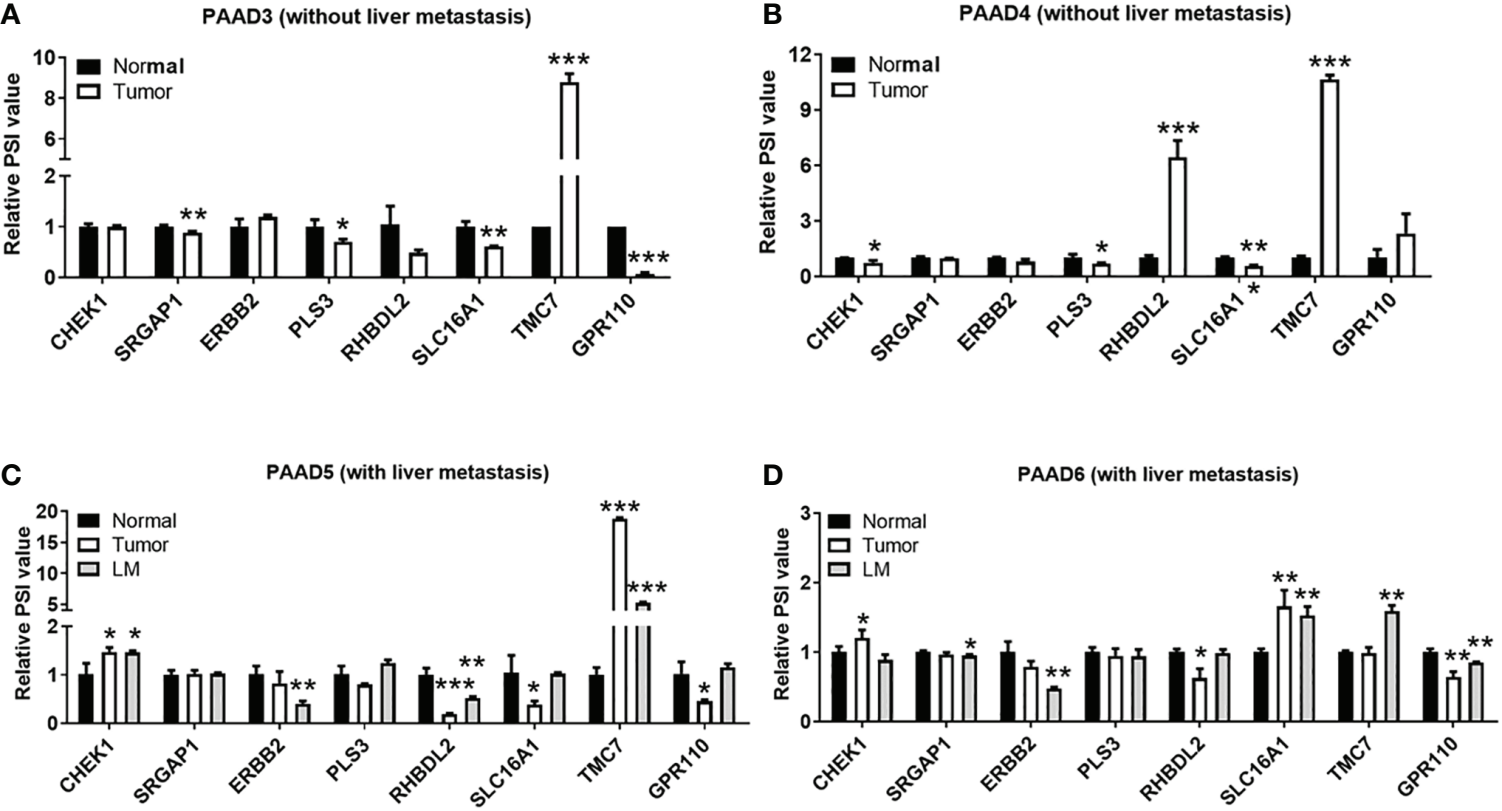
Validation of gene AS events associated with liver metastases in pancreatic cancer. By qRT-PCR analysis, expression of right gene AS events in pancreatic cancer tissues without liver metastases **(A, B)** n = 2 and pancreatic cancer with hepatic metastases **(C, D)** n = 2. AS, alternative splicing. P value, "*" 0.01-0.05; "**" 0.001-0.01; "***" 0_0.001.

As shown in **Figure 4**, qRT-PCR analysis was performed to explore the relationship between these AS events and the liver metastasis of pancreatic cancer. The expression of eight AS events in pancreatic cancer tissues without liver metastases (n = 2) or with hepatic metastases (n = 2) is exhibited in **Figures 4A, B**. AS events of TMC7 and RHBDL2 showed higher expression in the primary PDAC tumor without liver metastasis (**Figures 4A, B**). However, AS events of TMC7 and CHEK1 showed higher expression in primary and metastatic PDAC tumors with liver metastasis (**Figures 4C, D**).

### 3.5 SiRNA targeting exon 17 of TMC7 inhibits invasion and migration of pancreatic cancer cells

**Figure 5:**
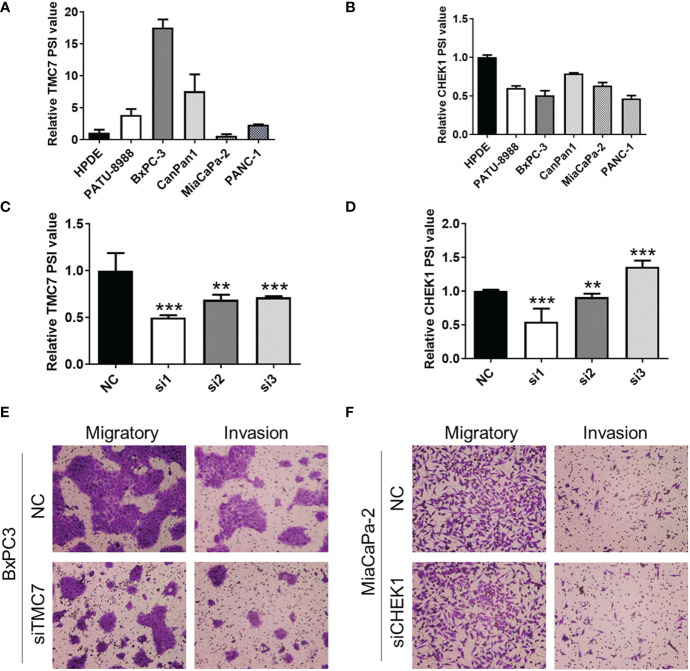
Knockdown of exon 17 of TMC7 inhibits invasion and migration of pancreatic cancer cells. **(A)** Expression of TMC7 AS events in pancreatic cancer cell lines and normal pancreatic cell lines by qRT-PCR. **(B)** After treatment by siRNAs targeting TMC7 exon 17, RNA levels of TMC7 exon 17 and exon 16 were detected by qRT-PCR. **(C)** After treatment by siRNAs targeting TMC7 exon 17, migratory and invasion abilities were assessed. **(D)** Expression of CHEK1 AS events in pancreatic cancer cell lines and normal pancreatic cell lines by qRT-PCR. **(E)** After treatment by siRNAs targeting CHEK1 exon 13.3, RNA levels of CHEK1 exon 13.3 and exon 13.1 were detected by qRT-PCR. **(F)** After treatment by siRNAs targeting CHEK1 exon 13.3, migratory and invasion abilities were evaluated. AS, alternative splicing. P value, “**” 0.001-0.01; “***” 0_0.001.

We evaluated the expression of TMC7 and CHEK1 AS events in pancreatic cancer cell lines and normal pancreatic cell lines by qRT-PCR. Then, we selected cell lines with high expression of TMC7 (BxPC-3) and CHEK1 (CanPan1) AS events for the next experiments (**Figures 5A, B**). Then, we designed siRNA sequences especially targeting exon 17 of TMC7 and exon 13.3 of CHEK1. These siRNA sequences successfully decreased the PSI value of TMC7 and CHEK1 (**Figures 5C, D**). By Transwell assays, siRNA targeting on exon 17 of TMC7 could apparently suppress the migration and invasion of PDAC cancer cells (**Figure 5E**). In contrast, the siRNA sequences targeting exon 13.3 of CHEK1 exhibited limited influence on the migration and invasion of PDAC cancer cells (**Figure 5F**).

### 3.6 Exon 17 of TMC7 promotes invasion of patient-derived organoids and is associated with poor prognosis in PDAC

**Figure 6:**
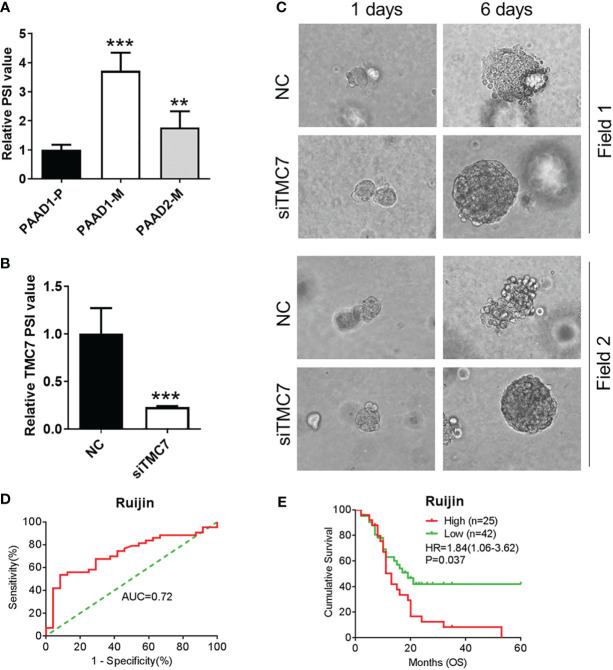
Exon 17 of TMC7 regulates invasion of PDAC-derived organoids and is significantly associated with inferior prognosis in PDAC. **(A)** Expression of TMC7 AS events in pancreatic cancer organoids derived from primary or liver metastatic tissues. **(B)** After treatment by siRNAs targeting TMC7 exon 17, RNA levels of TMC7 exon 17 and exon 16 were detected by qRT-PCR. **(C)** After treatment by siRNAs targeting TMC7 exon 17 for 6 days, the invasion ability of organoids was evaluated by 3D invasion assay. **(D)** A ROC curve was plotted to acquire optimal cutoff value to predict patients’ overall survival. **(E)** Kaplan–Meier survival curves of TMC7 PSI value in PDAC. PDAC, pancreatic ductal adenocarcinoma; AS, alternative splicing; ROC, receiver operating characteristic. P value, “**” 0.001-0.01; “***” 0_0.001.

We successfully established a primary tumor-derived PDAC organoid and two liver metastatic PDAC tumor-derived organoids (**Supplementary Figure 1**). By qRT-PCR, we found that metastatic organoids showed a higher level PSI value of TMC7 (**Figure 6A**). Then, we used siRNA to successfully decrease the PSI value of TMC7 in metastatic organoids. Additionally, the siRNA can inhibit the invasion ability of organoids, according to the 3D invasion assay (**Figures 6B, C**). Finally, we further calculated the TMC7 PSI value in 67 PDAC cases. By plotting the ROC curve, an optimal cutoff value was set (**Figure 6D**). High expression of TMC7 PSI value was significantly involved in poor prognosis in PDAC (**Figure 6E**, p = 0.037).

## 4 Discussion

Pancreatic cancer is a highly aggressive and metastatic disease with a 5-year overall survival rate of 10% (1). After surgery, approximately 60% of PDAC patients have distant metastasis within the first 24 months (23), which is one of the primary causes of mortality in these patients (24). In the process of PDAC metastasis, EMT plays a crucial role (25). Recently, the roles of gene alternative splicing in pancreatic cancer have been proved, such as carcinogenesis and metastasis (26, 27). In this study, we redefined the gene set associated with pancreatic cancer EMT, which was validated in other datasets, PDAC organoids, and our clinical center. Furthermore, we applied this gene set for further analysis, and eight key AS events associated with pancreatic cancer progression were identified. Among them, we found that TMC7 and CHECK1 AS events were increased in metastatic lesions and pancreatic cancer liver tissue-derived organoids. Moreover, we found that the knockdown of exon 17 on TMC7 significantly inhibited the invasion, migration, and proliferation of the cell line and organoid model. Additionally, the expression of exon 17 on TMC7 was closely positively correlated with the progression of pancreatic cancer. Therefore, we found that exon 17 of TMC7 is a potential target in treating pancreatic cancer liver metastasis, according to a series of bioinformatics analyses and *in vitro* experiments.

The transition from epithelial to mesenchymal is a process where cells acquire mesenchymal characteristics with the ability to migrate (25). When researchers performed EMT-related bioinformatics analysis, they used different EMT-related gene sets (18, 19). In this study, we first downloaded two EMT-related gene sets that positively or negatively regulate the EMT process. We found that the enrichment scores of these two EMT gene sets were positive, which indicated that it is inappropriate to directly use these two gene sets when the researchers were performing bioinformatics analysis in PDAC. Interestingly, not all genes were significantly associated with prognosis, and some genes even exhibited an opposite relationship with prognosis according to the prognosis analysis. Therefore, 13 genes that positively regulated EMT were redefined as the EMT-related gene set in PDAC for the next analysis, which was further validated in another dataset, the Ruijin cohort and organoid model.

TCGA database is a suitable platform for mining the gene alternative splicing involved in carcinogenesis and cancer progression (14). Markolin et al. used HIF-dependent alternative splicing events as a clue to identify hypoxia-driven AS events (28). According to the 13 genes on the EMT-related gene set, we screened the gene AS events. Furthermore, eight critical gene AS events were identified in the Ruijin cohort and organoid model. The upregulation of TMC7 and CHECK1 AS events was observed in liver metastatic tissues according to the qPR-PCR results. Then, we further knocked down the expression of exon 17 on TMC7 and exon 13.3 on CHECK1 in the PDAC cell line and organoid. Suppressed invasion and migration of PDAC cases can only be observed on the cells and organoids treated with siRNA targeting exon 17 on TMC7. According to the informatics analysis, Cheng et al. proved that the expression of TMC7 mRNA was significantly related to the poor prognosis of PDAC. Furthermore, the knockdown of TMC7 mRNA could effectively suppress the clonability and invasiveness of PDAC cells (29). Additionally, TMC7 could also induce cancer cell proliferation and metastasis in oral tongue squamous cell carcinoma (30).

However, the mechanisms of how TMC7 promotes cancer development are still unclarified. Our result further revealed the critical role of exon 17 on TCM7 in regulating the development of PDAC.

## 5 Conclusion

In conclusion, according to a series of bioinformatics analysis methods, external validation, and experiments on *in vitro* cell line models and organoid models, we redefined the EMT-related gene set involved in PDAC liver metastasis. Furthermore, we also identified AS event of TMC7 as a crucial role in PDAC metastasis. Finally, we indicated that exon 17 on TCM7 could be a potential therapeutic target for treating pancreatic cancer liver metastasis.

## Data availability statement

The datasets presented in this study can be found in online repositories. The names of the repository/repositories and accession number(s) can be found below: https://figshare.com/s/f134598224000856ce3d.

## Ethics statement

All patients signed the informed consent form prior to participation in the study. The usage of tissues in this study was approved by the Institutional Review Board of Ruijin Hospital Affiliated to Shanghai Jiaotong University School of Medicine.

## Author contributions

YW, HQ, and LH performed the experiment. YW and HQ contributed significantly to analysis and manuscript preparation. YW, HQ, and LH performed the data analyses and wrote the manuscript. SZ helped perform the analysis with constructive discussions. XD and BS contributed to the design of the study and were responsible for the conception of the study. All authors contributed to the article and approved the submitted version.
