# Supplementary material for: Identification of EMT-related alternative splicing event of TMC7 to promote invasion and migration of pancreatic cancer
Source: Front Immunol. 2023 Jan 12;13:1089008. doi: 10.3389/fimmu.2022.1089008 (PMC9878378; doi:10.3389/fimmu.2022.1089008)
Supplement: Supplementary file 1 [file DataSheet_1.docx]

Supplementary Material

# Supplementary Tables

**Supplementary Table 1.** Sixty-seven PAAD patients included for prognosis analysis in this study.

| Age (years) | Count(n) | Percentage (%) |
| --- | --- | --- |
| ≤60 | 24 | 35.8 |
| ＞60 | 43 | 64.2 |
| Gender |  |  |
| Male | 26 | 38.8 |
| Female | 41 | 61.2 |
| Tumor size |  |  |
| ≤4 cm | 53 | 79.1 |
| >4 cm | 14 | 20.9 |
| T stage |  |  |
| T1 | 8 | 11.9 |
| T2 | 20 | 29.9 |
| T3 | 28 | 41.8 |
| T4 | 11 | 16.4 |
| Lymph node metastasis |  |  |
| Yes | 24 | 35.8 |
| No | 43 | 64.2 |
| Distant metastasis |  |  |
| Yes | 55 | 82.1 |
| No | 12 | 17.9 |
| Differentiation |  |  |
| Well/moderate | 21 | 31.3 |
| Poor | 46 | 68.7 |
| TNM stage |  |  |
| I | 8 | 12.0 |
| II | 37 | 55.2 |
| III | 10 | 14.9 |
| IV | 12 | 17.9 |

**Supplementary Table 2**. The primer sequences and siRNA sequences used in this study.

| **CTNNB1** | FORWARD | TGGATTGATTCGAAATCTTGCC |
| --- | --- | --- |
|  | REVERSE | GAACAAGCAACTGAACTAGTCG |
| **SMAD3** | FORWARD | AGAGAGTAGAGACACCAGTTCT |
|  | REVERSE | GAAGTTAGTGTTTTCGGGGATG |
| **EZH2** | FORWARD | AAATCAGAGTACATGCGACTGA |
|  | REVERSE | GTATCCTTCGCTGTTTCCATTC |
| **GCNT2** | FORWARD | GGAAAGATTTTTGGAAGCAGCT |
|  | REVERSE | AAACAGGCTTGGTGAATTAACC |
| **TCF7L2** | FORWARD | AAAGTGCGTTCGCTACATACAA |
|  | REVERSE | GAGGCGAATCTAGTAAGCTTCC |
| **SDCBP** | FORWARD | TTCTGCTCCTATCCCTCACGATGG |
|  | REVERSE | CTTGCTACCAACTGCCCCTGAAG |
| **WWTR1** | FORWARD | GATGGAGAGAGAAAGGATTCGA |
|  | REVERSE | TCCATTGAGGAAAGGATCTGAG |
| **BCL9L** | FORWARD | CTGTCTCCTCGTGTCCGCATTG |
|  | REVERSE | CAGCCACATCAGCAGGGAAAGG |
| **BMP2** | FORWARD | GACGTTGGTCAACTCTGTTAAC |
|  | REVERSE | GTCAAGGTACAGCATCGAGATA |
| **TWIST1** | FORWARD | GTACATCGACTTCCTCTACCAG |
|  | REVERSE | CATCCTCCAGACCGAGAAG |
| **TGFB2** | FORWARD | GCAAAGTTGTGAAAACAAGAGC |
|  | REVERSE | ATCCCAGGTTCCTGTCTTTATG |
| **COL1A1** | FORWARD | AAAGATGGACTCAACGGTCTC |
|  | REVERSE | CATCGTGAGCCTTCTCTTGAG |
| **SERPINB3** | FORWARD | AGATTAACTCCTGGGTGGAAAG |
|  | REVERSE | CAATGTGGTATTGCTGCCAATA |
| **CHEK1-13.1** | FORWARD | TAAAGGGAAGCTGATTGATATTGTG |
|  | REVERSE | GATGGTCCGATCATGTGGC |
| **CHEK1-13.3** | FORWARD | TTTACCTGCTTTACATTTCC |
|  | REVERSE | TCCCACCATTCTATACAAAT |
| **ERBB2-6** | FORWARD | ACAGGAGAAGGAGGAGGTGGAGG |
|  | REVERSE | GGCAAGAGGGCGAGGAGGAG |
| **ERBB2-14/15** | FORWARD | AGGTGAGGGCAGTTACCAGTG |
|  | REVERSE | AAATGCCAGGCTCCCAAAG |
| **GPR110-13** | FORWARD | CTCACTTGACCTCCTTCTCC |
|  | REVERSE | TGTGGCACCAACAATAAAC |
| **GPR110-17** | FORWARD | AGAAACCTCCGTGTCCAAG |
|  | REVERSE | TCCCAGTCGCCTACAATAA |
| **PLS3-5** | FORWARD | CGGAGTTTCACCGTGTTAG |
|  | REVERSE | GACTGGAGGTTGCTGCTTT |
| **PLS3-6** | FORWARD | AGCAACGGATTCATTTGTG |
|  | REVERSE | TTCCCATCTTTATTCCTGTCA |
| **RHBDL2-3** | FORWARD | GCCTCAGAAATCCTAAGCAA |
|  | REVERSE | ATCCCACTACAGACATTAACCAT |
| **RHBDL2-4** | FORWARD | GAGATGAAAGAAGAGCTGGAGG |
|  | REVERSE | GGAGATGATGAACACGGGAG |
| **SLC16A1-5.1** | FORWARD | CAGGGAAAGATAAGTCTAAAGC |
|  | REVERSE | AAAGCCTCTGTGGGTGAAT |
| **SLC16A1-6** | FORWARD | GGCATCATGGCAGATAGAG |
|  | REVERSE | AGAGGTTACAGACTTGGGT |
| **SRGAP1-1/2.2** | FORWARD | TGCCCATTGAAAGCAAACC |
|  | REVERSE | AGCTGAACTCGCATCTCCGT |
| **SRGAP1-2.2/3** | FORWARD | CAAACGGAGATGCGAGTTC |
|  | REVERSE | TTGGTTCAGGAGCAAATACC |
| **TMC7-16.2** | FORWARD | GGGACATTTACCCAGGACA |
|  | REVERSE | CAGGCATGAGCCACCACA |
| **TMC7-17** | FORWARD | GTTTACCTAACCCAGCCCTTAA |
|  | REVERSE | CGTCTCCGCTCTTTCATTCT |
| **TMC7-17 siRNA** |  |  |
| **si1** | FORWARD | GCCCAUGAGUGUAUAACAATT |
|  | REVERSE | UUGUUAUACACUCAUGGGCTT |
| **si2** | FORWARD | GGAGUAGCCUGGAAUUUAATT |
|  | REVERSE | UUAAAUUCCAGGCUACUCCTT |
| **si3** | FORWARD | GCAGCUCUCUCUAACAAAUTT |
|  | REVERSE | AUUUGUUAGAGAGAGCUGCTT |
| **CHEK1-13.3 siRNA** |  |  |
| **si1** | FORWARD | CCUCUUCUGUAAGCUCUAATT |
|  | REVERSE | UUAGAGCUUACAGAAGAGGTT |
| **si2** | FORWARD | GUGGGAAUUUGUAUAGAAUTT |
|  | REVERSE | AUUCUAUACAAAUUCCCACTT |
| **si3** | FORWARD | GCCUCAGUAAAGACACUGATT |
|  | REVERSE | UCAGUGUCUUUACUGAGGCTT |
