# Supplementary material for: Identification of EMT-related alternative splicing event of TMC7 to promote invasion and migration of pancreatic cancer
Source: Front Immunol. 2023 Jan 12;13:1089008. doi: 10.3389/fimmu.2022.1089008 (PMC9878378; doi:10.3389/fimmu.2022.1089008)
Supplement: Supplementary file 2 [file DataSheet_2.docx]

Supplementary Material

# Supplementary Figures
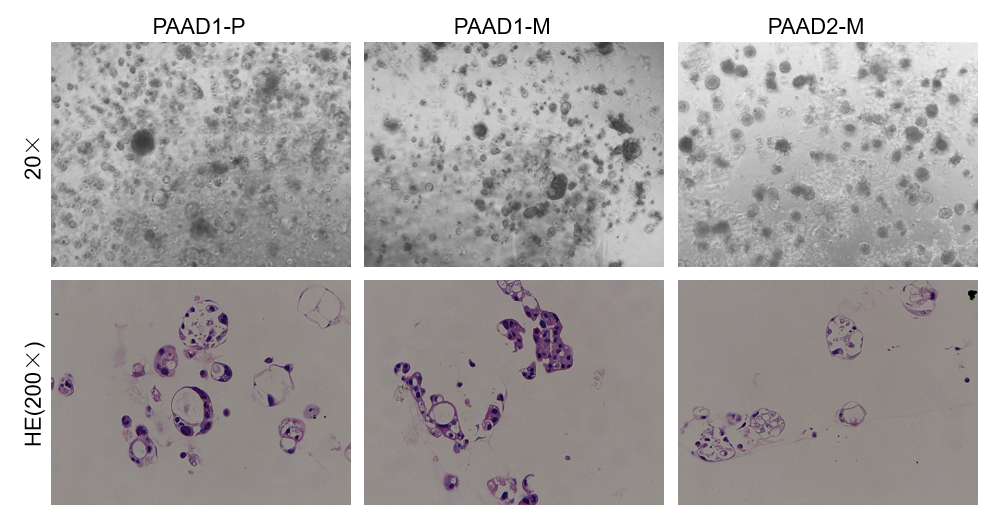


**Supplementary Figure 1.** PDAC-derived organoids are constructed in this study. Two primary PDAC-derived organoids and one PDAC organoid derived from liver metastatic tissue are constructed and verified by HE staining.
